# Beware of using surrogates to investigate threatened species!

**DOI:** 10.1093/conphys/coaf001

**Published:** 2025-01-05

**Authors:** Manuel E Coffill-Rivera

**Affiliations:** Stokes School of Marine and Environmental Sciences, University of South Alabama, 600 Clinic Dr, Mobile, AL 36688, USA; Dauphin Island Sea Lab, 101 Bienville Blvd, Dauphin Island, AL 36528, USA

If we are to save threatened species, we need to know much more about how they are functioning in a rapidly changing world. But studying a threatened species is not easy. Most of the time they are rare and hard to find, and it can be costly or challenging to make measurements. One solution is to draw conclusions by studying a substitute, or surrogate, species, that is closely related and easier to work with. But is it likely that the threatened species will respond in the same way as the investigated surrogate?

Not so, according to Jessica Reemeyer and colleagues, who investigated the effects of warming waters and reduced oxygen levels on the functioning of two morphologically and ecologically similar fish species: the Pugnose Shiner (*Miniellus anogenus*), listed as threatened, and the non-imperilled Blackchin Shiner (*Miniellus heterodon*) (Fig. 1).

**Figure 1:**
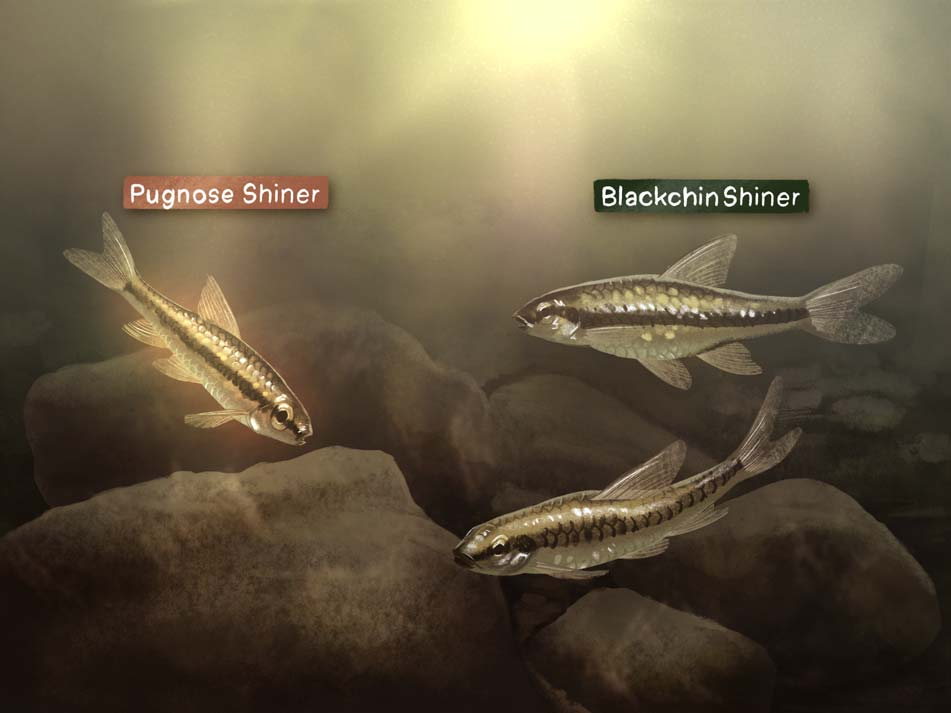
Illustration: Kaitlin Barham (kaitlin.barham@student.uq.edu.au)

The researchers collected individuals of both species from the same water body in Canada over 5 months, to conduct experiments in which they made standard measurements to understand how much heat and how little oxygen the fish could cope with.

Unsurprisingly, they were only able to collect about half as many of the threatened species as compared to the surrogate. Nevertheless, they had enough fish to draw strong conclusions about differences in how the two species responded. The Pugnose Shiner displayed lower tolerance to high water temperatures and low oxygen conditions than the Blackchin Shiner. When dissolved oxygen levels were low, the Pugnose Shiner’s ability to withstand high water temperatures decreased even further, while the Blackchin Shiner was unaffected. While the Blackchin Shiner showed the ability to adapt to varying oxygen levels, the Pugnose Shiner exhibited much less adaptability.

Reemeyer’s team has shown that how species respond to changing environmental conditions can differ widely, even among closely related species. If inferences about the threatened species had been drawn using the surrogate species, the conclusion would be that they are much more tolerant of increases in temperature and reduced oxygen availability than they actually are. While it might seem intuitive to make generalizations across species within the same genus, the team’s findings reveal how important it is to consider differences at the species level.

This specificity matters when managing and conserving at-risk species, particularly as climate change continues to alter their habitats. Using surrogates requires thorough validation to ensure that the responses truly reflect those of the imperilled species. Careful application of this knowledge can make the difference between meaningful conservation outcomes and misguided management decisions.

## Notes

**Editor:** Ashleigh Donaldson
